# Prevalence of falls in a pooled US cohort of adults with systemic lupus erythematosus: a nested cross-sectional study

**DOI:** 10.1136/lupus-2026-002156

**Published:** 2026-08-17

**Authors:** Laura C Plantinga, Jessica Fitzpatrick, S Sam Lim, Maria Dall’Era, Charmayne Marie Dunlop-Thomas, Courtney Hoge, Patricia P Katz, Jinoos Yazdany, C Barrett Bowling

**Affiliations:** 1 Department of Medicine, University of California, San Francisco, San Francisco, California, USA; 2 Department of Epidemiology and Statistics, University of California, San Francisco, San Francisco, California, USA; 3 Department of Medicine, Emory University, Atlanta, Georgia, USA; 4 Department of Epidemiology, Emory University, Atlanta, Georgia, USA; 5 Department of Behavioral, Social, & Health Education Sciences, Emory University, Atlanta, Georgia, USA; 6 Department of Medicine, Duke University, Durham, North Carolina, USA; 7 Geriatrics Research, Education, and Clinical Center, Durham Veterans Affairs Health System, Durham, North Carolina, USA

**Keywords:** Epidemiology, Lupus Erythematosus, Systemic, Patient Reported Outcome Measures, Prevalence, Risk Factors

## Abstract

**Objective:**

Epidemiologic data on falls in the population with systemic lupus erythematosus (SLE) remain sparse. We estimated the prevalence and correlates of falls among adults with SLE and compared SLE prevalence estimates with those in the general US population.

**Methods:**

We assessed falls in a nested cross-sectional study within two pooled population-based SLE cohorts in California (3/2025–2/2026) and Georgia (10/2019–5/2022). Stratified prevalence was assessed with marginal estimates from logistic regression models with falls as the outcome. Age-standardised and sex-standardised estimates in SLE were compared with 2023 US population estimates (ages ≥45 only).

**Results:**

In this pooled SLE cohort (N=780; mean age, 47.9; 91.9% women; 14.5% Asian, 51.7% black, 13.2% Hispanic), 26.0% reported any fall in the prior year; among these, 59.1% reported falling two times or more and 31.0% reported associated injuries. General factors (oldest vs youngest age (37.4% vs 20.7% for ≥65 vs 20–44)) and SLE-related factors (higher SLE activity (38.5% vs 13.5%), moderate-to-severe depressive symptoms (40.0% vs 23.7%), greater pain interference (43.8% vs 16.5%), greater fatigue (39.2% vs 18.8%) and lower cognitive function (39.7% vs 18.9%)) were statistically significantly associated with higher fall prevalence in the SLE cohort. Among those aged ≥45, the age-standardised and sex-standardised prevalence estimate in the SLE population (22.9%) was similar to the US population estimate (19.5%).

**Conclusion:**

The prevalence of falls in SLE is high and recurrence is common, suggesting clinical assessment of falls—especially in those with high disease activity and symptoms—is important in SLE.

**WHAT IS ALREADY KNOWN ON THIS TOPIC**

- Recent estimates suggest that about one-third of adults with systemic lupus erythematosus (SLE) fall over the course of a year and that those with higher disease activity report a higher prevalence of falls.

**WHAT THIS STUDY ADDS**

- This study pools data from two diverse adult SLE cohorts to provide more generalisable estimates of the prevalence of falls and compares these estimates with those in the similarly aged US population.

**HOW THIS STUDY MIGHT AFFECT RESEARCH, PRACTICE OR POLICY**

- Given the high prevalence, recurrence and SLE-specific correlates of falls that we observed in this study, clinical assessment of falls remains an important component of holistic SLE care, especially for patients with high disease activity, pain, fatigue, depressive symptoms or cognitive complaints.

## Introduction

 With both greater life expectancy due to advances in treatments for systemic lupus erythematosus (SLE)^1^ and greater proportions of individuals newly diagnosed with SLE at older ages (15% diagnosed after age 60),^2^ this population is ageing. Furthermore, regardless of age, widespread chronic inflammation and related symptoms across multiple organ systems in the setting of SLE may contribute to accelerated ageing among those with this disease.^3 4^ These factors and associated frailty^5 6^ may put those with SLE at increased risk of outcomes associated with older age, such as falls and associated injuries. However, data on falls in SLE remain sparse.^7^

Recently, we showed that nearly one-third of participants in a large, population-based US adult SLE cohort (Georgians Organized Against Lupus (GOAL)) reported falling in the past year, and that disease activity and damage, but not age or sex, were associated with a higher prevalence of falls.^8^ However, gaps remain in our knowledge of the epidemiology of falls in SLE, including whether these results generalise to a larger, more representative and more diverse cohort and how the prevalence of falls in the SLE population compares to that in the similarly aged US population. To address these gaps, we pooled the previously published GOAL data^8^ with newly collected data from the California Lupus Epidemiology Study (CLUES) to estimate the prevalence and correlates of falls among adults with SLE and compare fall prevalence estimates in SLE to those in the general US population.

## Methods

### Data sources and study populations

#### Study population

CLUES and GOAL are ongoing, population-based cohorts that enrol adults (≥18 years) living in the San Francisco Bay Area and metropolitan Atlanta, respectively, with a documented diagnosis of SLE defined by American College of Rheumatology (ACR) criteria^9^ (4 ACR criteria, or 3 ACR criteria with a diagnosis of SLE by an attending board-certified rheumatologist) or, in CLUES, by a diagnosis of lupus nephritis.^10^ In this nested cross-sectional study, data were obtained via interviewer-administered questionnaire as part of the 10th annual CLUES survey (3/2025–2/2026; ‘CLUES Time 10’) and via self-administered questionnaire in a cross-sectional ancillary study to GOAL (Approaches to Positive, Patient-centered Experiences of Aging with Lupus (APPEAL); 10/2019–5/2022) (online supplemental figure 1).^11^ CLUES Time 10 and APPEAL participants with incomplete falls questionnaires or potentially invalid surveys (identified by identical responses on reverse-coded items) were excluded; those aged <45 were additionally excluded for comparisons with the US population (online supplemental figure 2). The University of California San Francisco Institutional Review Board approved the CLUES study (14–14429), and the Emory University Institutional Review Board approved the GOAL (IRB00003656) and APPEAL (IRB00110977) study protocols. All CLUES, GOAL and APPEAL participants provided informed consent prior to participation.

#### US population

We obtained the most recent available data on US fall prevalence from the 2023 Behavioral Risk Factor Surveillance Survey (BRFSS; combined landline and cell phone survey data), available from the National Center for Health Statistics (online supplemental figure 1).^12^ Data on falls were collected only for adults aged 45 years or older in the BRFSS (online supplemental figure 2). We used the 2020 American Community Survey 5-year estimate files, available from the US Census Bureau (online supplemental figure 1),^13^ to obtain US population census data, stratified by age, sex and race/ethnicity,

### Variables

#### Falls

The number of falls in the prior year and the number of falls requiring medical attention in the prior year were collected for each participant at a single time point in CLUES, GOAL and BRFSS, with minor differences in the wording of questionnaire items across the data sources (online supplemental table 1). ‘Any falls’ was defined as ≥1 fall reported in the prior year; ‘two or more falls’ was defined as ≥2 falls reported in the prior year; and ‘any injury fall’ was defined as ≥1 fall that limited activities (BRFSS) or required medical attention (all data sources) in the prior year.

#### Other variables

Other variables of interest included demographics, SLE-related factors and other clinical and psychosocial factors.

### Demographics

Demographic variables of interest included age, sex (assigned at birth), race and ethnicity. Race and ethnicity were self-reported at the time of falls assessment by participants from a fixed set of categories (American Indian/Alaska Native, Asian, black, Hawaiian or Pacific Islander, and white for race; Hispanic and non-Hispanic for ethnicity). These variables were then combined and categorised as follows to align with the national data: Asian (non-Hispanic), black (non-Hispanic), Hispanic (regardless of race), white (non-Hispanic) and other.

### SLE-related factors

SLE duration was self-reported. For GOAL, duration was taken from the closest GOAL survey to the APPEAL assessment and adjusted for the time between measurements (median, 2.7 months; online supplemental figure 1). SLE activity was assessed at the time of falls assessment in both CLUES and GOAL, using the Systemic Lupus Activity Questionnaire (SLAQ; range 0–44; higher scores indicating greater SLE-related disease activity).^14^ Cumulative SLE-related damage to individuals’ systems was assessed by the Brief Index of Lupus Damage (BILD) score (range, 0–46; higher scores indicate greater cumulative SLE-related organ damage).^15 16^ BILD was not assessed simultaneously with falls in either cohort. For CLUES, we used the BILD score collected in the previous assessment (time 9, 2/2024–3/2025); for GOAL, we used the BILD score in the annual GOAL survey closest to the participant’s APPEAL visit (online supplemental figure 1).

### Other clinical and psychosocial variables

Clinical and psychosocial factors of interest included body mass index (BMI), physical activity, physical functioning, depressive symptoms, pain interference, fatigue, sleep disturbance, cognitive function, perceived stress and opioid use. BMI was assessed by measured (APPEAL in-person visits) or self-reported (CLUES interviews and APPEAL remote visits) height and weight; BMI was categorised as obese (≥30 kg/m^2^) versus not (<30 kg/m^2^). In CLUES, physical activity was assessed with the item ‘Which of the following best describes your physical activity level?’ with possible responses of: ‘seldom active, rarely active’ (categorised as low), ‘somewhat active for at least 30 min, 3 or more times a week’ (categorised as moderate/high) and ‘very active for at least 30 min, 3 or more times a week’ (categorised as moderate/high). In GOAL, physical activity was measured with the International Physical Activity Questionnaire Short Form (IPAQ-SF) at the APPEAL visit, and activity level was collapsed as low and moderate/high, to match the CLUES categories.^17^ Perceived physical function was assessed with Patient-Reported Outcomes Measurement Information System (PROMIS) Physical Functioning-Short Forms (CLUES: V.10a; GOAL, V.12a), with raw scores scaled to comparable T-scores (population mean=50, SD=10).^18 19^ Depressive symptoms were assessed with the 8-item Patient Health Questionnaire (PHQ-8; score range, 0–12)^20^ and the PROMIS Depression Short Form 8^21^ in CLUES and GOAL, respectively, with higher scores indicating a greater burden of depressive symptoms in both assessments. Cut-offs of ≥10^20^ and ≥58.8^22^ for the PHQ-8 score and PROMIS Depression Short Form 8b T-score were used to define moderate to severe versus no or mild depressive symptoms. PROMIS Pain Interference, Fatigue and Sleep Disturbance Short Forms were used in both CLUES (V.4a) and GOAL (V.8a) to measure pain interference (the extent to which pain affects engagement with social, cognitive, emotional, physical and recreational activities), fatigue and sleep disturbance, respectively; comparable T-scores were reported, with higher scores indicating higher pain interference, fatigue and sleep disturbance. Similarly, perceived cognitive function was assessed with the PROMIS Cognitive Function Short Form in CLUES (V.6a) and GOAL (V.8a), with higher T-scores, indicating higher perceived function. In CLUES, pain interference, fatigue, sleep disturbance and cognitive function measures were measured simultaneously with falls, while in GOAL, these variables were taken from the closest GOAL survey to the APPEAL ancillary study (online supplemental figure 1). The Perceived Stress Scale (PSS) score^23 24^ (scale, 0–16; higher scores indicate higher perceived stress) was measured at the time of falls assessment in CLUES (4-item PSS; PSS-4) and APPEAL (10-item PSS; PSS-10); the relevant PSS-10 items were used to calculate the PSS-4. Use of opioid medications was self-reported by participants in both cohorts, also at the time of falls assessment.

### Patient and public involvement

Patients were involved in the design and conduct of this research. We reviewed unstructured feedback, provided directly to research coordinators during visits, from our pilot study of GOAL participants^25^ to create our initial protocol; similarly, participant feedback was reviewed during weekly research team meetings throughout the study to modify the protocol as needed. Participant burden was carefully considered in the number and order of measures assessed in both APPEAL and CLUES; participants could skip assessments and take breaks as needed. Finally, GOAL and CLUES participants are informed of study results through regular study newsletters, which are suitable for a non-specialist audience.

### Statistical analysis

Descriptive statistics were used to describe the demographic, clinical and psychosocial characteristics of the study population. The overall prevalence of any falls, two or more falls and any injury falls were estimated overall and by age, sex and race/ethnicity. Prevalence estimates of any falls stratified by SLE-related and other clinical and psychosocial characteristics were postestimation marginal probabilities following multivariable logistic regression with any falls as the outcome and participant characteristics as the exposures, with adjustment for age and demographics (age, sex and race/ethnicity). Finally, we obtained estimates of fall prevalence standardised to the age and sex distribution of the US population (from the ACS 2020 5-year estimates). Among those aged ≥45 years, these estimates were then compared with the overall and age-stratified, sex-stratified and race/ethnicity-stratified estimates of fall prevalence in the US population, which we obtained from 2023 BRFSS, using appropriate survey weights and procedures per BRFSS guidelines.^12^ In sensitivity analyses, we (1) compared participant characteristics and falls between the cohorts using χ^2^, *t*, and Wilcoxon rank-sum tests as appropriate; (2) estimated fall prevalence stratified by cohort, with assessment of potential effect modification using interaction terms; (3) estimated falls prevalence across multiply imputed datasets for variables of interest with >20% missing data, using models including the variable of interest and age, sex, race/ethnicity, SLE activity and disease duration to perform the imputation and (4) added race/ethnicity to the standardisation of fall prevalence estimates in comparative analyses with the US population. Complete case analysis was used unless otherwise noted. All analyses were performed with Stata V.19.5 (StataCorp, College Station, Texas).

## Results

### Characteristics of the study population

Among 334 CLUES Time 10 and 451 APPEAL participants, 5 were excluded due to incomplete falls questionnaires (n=1) and potentially invalid surveys (n=4), leaving N=780 for primary analyses. For comparisons with the US population, 333 additional individuals aged <45 were excluded, leaving N=447 (online supplemental figure 2).

The mean (SD) age of the pooled SLE cohort (N=780) participants was 47.9 (13.5) years; 57.3% of participants were aged ≥45 years, and 12.7% were aged ≥65 years (table 1). Most (91.9%) were women, while 14.5%, 51.7%, 13.7% and 17.7% were Asian, black, Hispanic and white, respectively. At the time of falls assessment, participants had had SLE for a median of 18.3 years, and the median SLAQ score was 10.0. Over one-third (38.0%) of participants were obese, and 50.7% reported low physical activity. Mean physical and cognitive function T-scores were 44.0 and 46.3, while pain interference, fatigue and sleep disturbance T-scores were 55.2, 55.4 and 53.2. Moderate or severe depressive symptoms were reported by 14.4%, and 11.3% reported current opioid medication use.

**Table 1:** Selected characteristics of pooled systemic lupus erythematosus cohort participants

Characteristic	Value
N	780
Sociodemographic	
Mean (SD) age in years	47.9 (13.5)
Age category, n (%)	
20–44	333 (42.7%)
45–54	202 (25.9%)
55–64	146 (18.7%)
≥65	99 (12.7%)
Sex at birth, n (%)	
Women	717 (91.9%)
Men	63 (8.1%)
Race/ethnicity, n (%)	
Asian	113 (14.5%)
Black	403 (51.7%)
Hispanic	103 (13.2%)
White	138 (17.7%)
Other	23 (3.0%)
Clinical, physical, and psychosocial	
Median (IQR) disease duration, years	18.3 (10.5, 26.5)
Median (IQR) SLAQ score	10.0 (5.0, 16.0)
Median (IQR) BILD score	2.0 (1.0, 4.0)
Mean (SD) BMI, kg/m^2^	28.6 (7.6)
Obese, n (%)	
Yes	292 (38.0%)
No	476 (62.0%)
Physical activity, n (%)	
Low	392 (50.7%)
Moderate/High	381 (49.3%)
Mean (SD) physical function T-score	44.0 (10.1)
Depression, n (%)	
Moderate or severe	110 (14.4%)
None or mild	653 (85.6%)
Median (IQR) perceived stress score	6 (3, 8)
Mean (SD) pain interference T-score	55.2 (10.3)
Mean (SD) fatigue T-score	55.4 (11.2)
Mean (SD) sleep disturbance T-score	53.9 (10.4)
Mean (SD) cognitive function T-score	46.3 (10.2)
Opioid pain medications, n (%)	
Yes	88 (11.3%)
No	690 (88.7%)

Missing values: ethnicity (GOAL, n=1; CLUES, n=1), BILD score (CLUES, n=44), SLE duration (GOAL, n=1) BMI/obesity (GOAL, n=11; CLUES, n=1), physical activity (GOAL, n=7), depressive symptoms score (GOAL, n=17), pain interference (GOAL, n=7), cognitive function (GOAL, n=309), fatigue (GOAL, n=1), perceived stress score (GOAL, n=2) and opioid pain medications (CLUES, n=2).

BMI, body mass index; CLUES, California Lupus Epidemiology Study; GOAL, Georgians Organized Against Lupus; IQR, interquartile (25th–75th percentile) range; SLAQ, Systemic Lupus Activity Questionnaire (range, 0–44; 44 is maximum activity).

The cohorts had similar sex distributions (92.2% and 91.7% women in CLUES and GOAL, respectively), but on average, CLUES participants were older than GOAL participants (mean age, 50.3 vs 46.2 years; online supplemental table 2). Race and ethnicity distributions also differed, with 31.8%, 23.4% and 30.0% of CLUES participants being Asian, Hispanic and White, and 81.4% of GOAL participants being black. CLUES versus GOAL participants had longer median disease duration (24.0 vs 15.0 years) but lower median SLAQ scores (7.0 vs 13.0); as well, they were less likely to be obese (25.9% vs 47.2%) or have low physical activity (20.1% vs 73.9%). CLUES participants had higher scores for physical function and lower scores for pain interference, fatigue, sleep disturbance and stress, but lower cognitive scores, compared with GOAL participants; percentages of participants reporting moderate-to-severe depressive symptoms or opioid use were similar across cohorts (online supplemental table 2).

### Prevalence and correlates of falls

Overall, 26.0%, 15.0% and 8.1% of the pooled SLE cohort participants reported at least one fall, two or more falls and at least one injury fall, respectively, in the prior year (table 2). Of those who fell, 120 (59.1%) reported falling two times or more, and 63 (31.0%) reported at least one injury with their fall(s). Reports of any falls were more common among those who were older (37.4% vs 20.7% for those aged ≥65 vs 20–44 years), female (27.1% vs 14.3% for women vs men) and black (31.0%) or white (31.9%), compared with those who were Hispanic (19.4%) or Asian (8.0%), although CIs for the middle age, sex and Hispanic, black and white groups overlapped. These patterns were similar for prevalence estimates for two or more falls and for injury falls (table 2).

**Table 2:** Prevalence of falls in the total pooled cohort overall and by age, sex and race/ethnicity

Demographic	Any fall in prior year		Two or more falls in prior year		Any fall with injury in prior year	
	n/N	% (95% CI)	n/N	% (95% CI)	n/N	% (95% CI)
Overall	203/780	26.0 (23.0 to 29.3)	120/780	15.4 (12.9 to 18.1)	63/780	8.1 (6.3 to 10.2)
Age						
20–44	69/333	20.7 (16.5 to 25.5)	38/333	11.4 (8.2 to 15.3)	15/333	4.5 (2.5 to 7.3)
45–54	54/202	26.7 (20.8 to 33.4)	37/202	18.3 (13.2 to 24.4)	18/202	8.9 (5.4 to 13.7)
55–64	43/146	29.5 (22.2 to 37.6)	23/146	15.8 (10.3 to 22.7)	16/146	11.0 (6.4 to 17.2)
≥65	37/99	37.4 (27.9 to 47.7)	22/99	22.2 (14.5 to 31.7)	14/99	14.1 (8.0 to 22.6)
Sex						
Women	194/717	27.1 (23.8 to 30.5)	115/717	16.0 (13.4 to 18.9)	61/717	8.5 (6.6 to 10.8)
Men	9/63	14.3 (6.8 to 25.4)	5/63	7.9 (2.6 to 17.6)	2/63	3.2 (0.4 to 11.0)
Race/ethnicity						
Asian	9/113	8.0 (3.7 to 14.6)	6/113	5.3 (2.0 to 11.2)	1/113	0.9 (0.02 to 4.8)
Black	125/403	31.0 (26.5 to 35.8)	77/403	19.1 (15.4 to 23.3)	35/403	8.7 (6.1 to 11.9)
Hispanic	20/103	19.4 (12.3 to 28.4)	9/103	8.7 (4.1 to 15.9)	5/103	4.9 (1.6 to 11.0)
White	33/138	31.9 (24.2 to 40.4)	27/138	19.6 (13.3 to 27.2)	20/138	14.5 (9.1 to 21.5)

Even after adjustment for age, sex and race/ethnicity, those with higher versus lower disease activity (30.4% vs 11.1%) and, to a lesser extent, cumulative disease damage (24.0% vs 15.9%, with overlapping CIs) had higher prevalence of any reported falls in the prior year (table 3). Sensitivity analyses using multiply imputed datasets for cumulative disease damage showed very similar results (age-adjusted, sex-adjusted and race/ethnicity-adjusted prevalence for high vs low disease damage: 22.7% (17.4%, 29.2%) vs 15.4% (11.3%, 20.6%)). After adjustment, obesity (21.0% vs 17.3%), low versus moderate–high physical activity (23.8 vs 16.3%) and sleep disturbance (24.4% vs 15.4%) were associated with higher prevalence of falls, but these differences were not statistically significant. Moderate-to-severe depressive symptoms (30.0% vs 16.9%) and higher perceived stress (24.7% vs 14.7%) remained associated with higher prevalence of falls, even after adjustment, with slight overlapping of CIs, as did higher pain interference (35.1% vs 13.8%), fatigue (29.4% vs 14.0%) and lower physical (34.2% vs 13.4%) function T-scores (all without overlap of CIs). Higher versus lower cognitive function was statistically significantly associated with lower adjusted prevalence of falls (14.6% vs 32.7%); sensitivity analyses using multiply imputed datasets to replace missing values for cognitive function were similar (15.4% (11.6%, 20.2%) vs 29.8% (22.1%, 38.9%)). Similarly, opioid use was statistically significantly associated with more than twofold higher prevalence of falls even after adjustment (40.9% vs 17.2%). These patterns were similar when examined by cohort; however, among those with lower disease activity (10.7% vs 17.9%), disease burden (12.1% vs 27.0%), pain interference (12.0% vs 21.3%) and fatigue (12.8% vs 24.3%), CLUES participants reported a lower prevalence of falls compared with GOAL participants (online supplemental table 3).

**Table 3:** Prevalence of any falls reported in the past year in the pooled cohort, by clinical and psychosocial characteristics

Characteristic	Percentage falling in prior year (95% CI)		
	Unadjusted	Age-adjusted	Age-adjusted, sex-adjusted and race/ethnicity-adjusted
Disease duration			
<10 years	24.1 (18.4 to 31.0)	27.9 (21.1 to 35.8)	19.9 (13.7 to 28.2)
≥10 years	26.6 (23.2 to 30.3)	25.1 (21.7 to 28.9)	18.8 (14.5 to 23.9)
Disease activity			
<Median (=9)	13.5 (10.5 to 17.4)	13.4 (10.3 to 17.1)	11.1 (7.9 to 15.4)
≥Median	38.5 (33.7 to 43.4)	38.0 (33.3 to 42.9)	30.4 (23.6 to 38.2)
Disease damage			
<Median (=2)	20.7 (16.8 to 25.1)	21.2 (17.2 to 25.7)	15.9 (11.6 to 21.5)
≥Median	32.3 (27.8 to 37.3)	31.2 (26.6 to 36.3)	24.0 (18.3 to 30.8)
Obesity			
No	22.7 (19.1 to 26.7)	22.4 (18.8 to 26.4)	17.3 (13.1 to 22.5)
Yes	31.5 (26.4 to 37.1)	31.0 (26.0 to 36.6)	21.0 (15.4 to 28.0)
Physical function			
High-average (T-score>40)	15.4 (12.4 to 18.9)	15.6 (12.5 to 19.2)	13.4 (9.9 to 17.8)
Low (T-score≤40)	42.0 (36.6 to 47.5)	41.0 (35.6 to 46.7)	34.2 (26.3 to 43.1)
Physical activity			
Low	31.9 (27.5 to 36.7)	32.0 (27.5 to 36.8)	23.8 (17.8 to 31.2)
Moderate–high	20.5 (16.7 to 24.8)	19.7 (16.0 to 24.0)	16.3 (12.2 to 21.6)
Depression			
Moderate or severe	40.0 (31.3 to 49.4)	39.7 (30.9 to 49.2)	30.0 (21.2 to 40.4)
None or mild	23.7 (20.6 to 27.2)	23.4 (20.2, 26.8)	16.9 (12.9 to 21.8)
Perceived stress			
<Median (=5)	19.3 (15.7 to 23.5)	18.4 (14.8 to 22.6)	14.2 (10.3 to 19.1)
≥Median	32.6 (28.2 to 37.5)	32.9 (28.4 to 37.8)	24.7 (19.0 to 31.5)
Pain interference			
High (T-score≥60)	43.8 (38.1 to 49.8)	43.2 (37.5 to 49.2)	35.1 (27.1 to 44.0)
Low-average (T-score<60)	16.5 (13.5 to 20.0)	16.4 (13.4 to 19.9)	13.8 (10.3 to 18.3)
Fatigue			
High (T-score≥60)	39.2 (33.6 to 45.1)	38.8 (33.2 to 44.8)	29.4 (22.5 to 37.3)
Low-average (T-score<60)	18.8 (15.6 to 22.4)	18.4 (15.3 to 22.1)	14.0 (10.3 to 18.5)
Sleep disturbance			
High (T-score≥60)	35.0 (28.0 to 42.7)	34.7 (27.7 to 42.5)	24.4 (17.2 to 33.5)
Low-average (T-score<60)	21.1 (17.6 to 25.0)	20.5 (17.1 to 24.4)	15.4 (11.3 to 20.7)
Cognitive function			
High-average (T-score>40)	18.9 (15.1 to 23.3)	18.3 (14.6 to 22.7)	14.6 (10.4 to 19.9)
Low (T-score≤40)	39.7 (31.2 to 48.8)	38.7 (30.2 to 48.0)	32.7 (23.3 to 43.6)
Opioid medication use			
Yes	52.2 (41.9 to 62.5)	50.7 (40.2 to 61.0)	40.9 (29.3 to 53.6)
No	22.8 (19.8 to 26.0)	22.6 (19.6 to 25.9)	17.2 (13.3 to 22.0)

### Comparison of fall prevalence in the SLE versus the US general population

**Figure 1:**
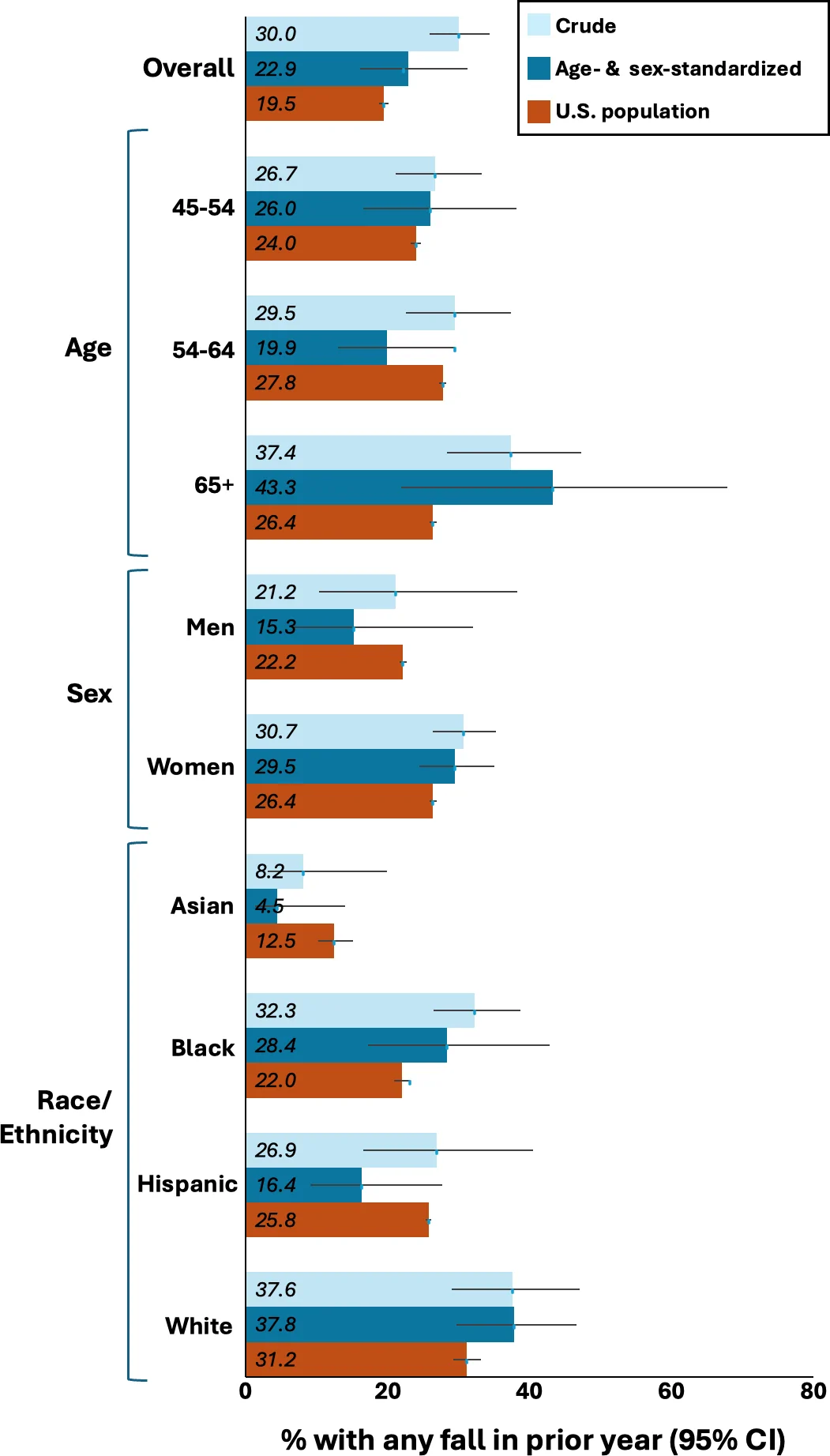
Crude and age-standardised and sex-standardised prevalence of any falls in the prior year among adults aged 45 and older in our pooled cohort with systemic lupus erythematosus vs in the US population. US population estimates were obtained using the 2023 Behavioral Risk Factors Surveillance Survey data.

Among pooled SLE cohort participants aged 45 and older, 30.0% reporting falling in the prior year; the age and sex-standardised estimate was 22.9%, similar to the estimate in the US population aged 45 years and older (19.5%; figure 1 and online supplemental table 4). Stratified, age-standardised and sex-standardised estimates from the SLE population were generally similar to the US population estimates; although estimates for the SLE versus US population were higher in the oldest age group (aged ≥65 years; 43.3% vs 25.4%) and among black (28.4% vs 22.0%) and white (37.8% vs 31.2%) subpopulations, the CIs of these estimates overlapped.

The age-standardised and sex-standardised prevalence of two or more falls in the pooled SLE cohort (16.0%) was higher than the US prevalence estimate (12.8%), but the CIs overlapped (online supplemental table 5). Those who were aged ≥65 years (27.4% vs 13.8%) or were white (32.1% vs 19.2%) had higher prevalence of two or more falls than their US general population counterparts. Overall, while not statistically significant, participants in the pooled SLE cohort reported fewer injury falls (age-standardised and sex-standardised estimate, 6.1%) than the US general population (10.0%); this pattern persisted across subgroups (online supplemental table 6).

**Figure 2:**
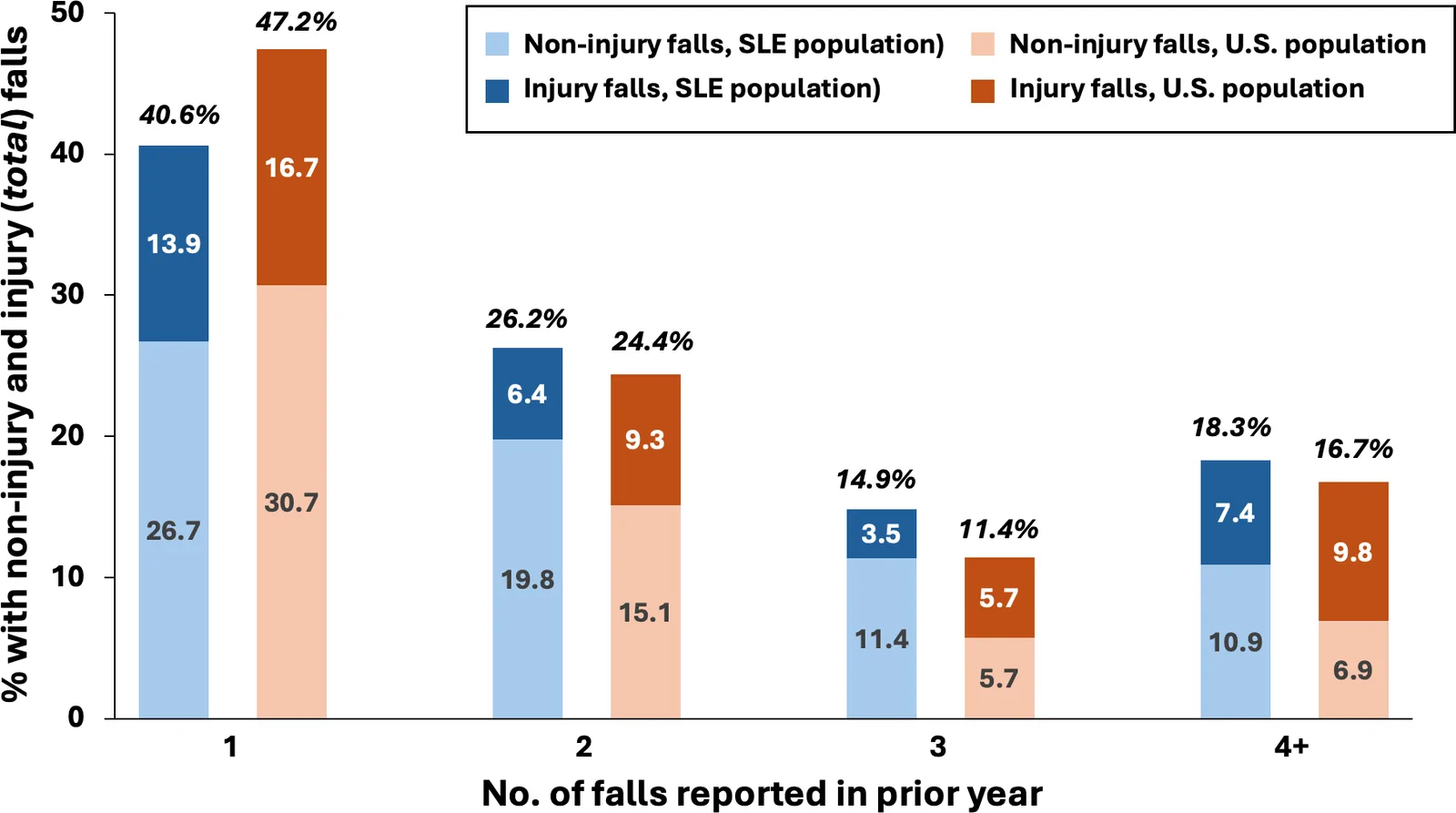
Distribution of number of falls and related injury in the pooled SLE cohort vs the US population, among those who reported falling in the prior year. US population estimates were obtained using the 2023 Behavioral Risk Factors Surveillance Survey data. SLE, systemic lupus erythematosus.

Among those in the pooled SLE cohort who were aged ≥45 years and reported any falls, 40.6%, 26.2%, 14.9% and 18.3% reported one, two, three and four or more falls (figure 2). These included 13.9%, 6.4%, 3.5% and 7.4% reporting at least one injury among their falls (representing 34.2%, 24.4%, 23.5% and 40.4% of those with one, two, three and four or more falls, respectively). In comparison, 47.2%, 24.2%, 11.4% and 16.7% of those in the US general population aged ≥45 years who reported any falls reported one, two, three and four or more falls (figure 2). These included 16.7%, 9.3%, 5.7% and 9.8% reporting at least one injury among their falls (representing 35.4%, 38.4%, 50.0% and 58.7% of those with one, two, three and four or more falls, respectively).

## Discussion

In this pooled diverse SLE cohort, about one-quarter of the adult participants reported falling in the prior year. Of these, more than half reported falling multiple times, and nearly one-third reported associated injuries. Higher fall prevalence was associated with greater overall disease activity and SLE symptoms (fatigue, pain interference, depressive symptoms and lower cognitive function) and older age in the pooled SLE cohort. Among SLE cohort participants aged 45 or older, about one-third reported falls, but the age-standardised and sex-standardised prevalence estimate was only slightly higher than the US population estimate.

Both this pooled-cohort study and our previous study in GOAL^8^ estimated a higher fall prevalence than a previous small cross-sectional study in Turkey, in which 15% of women with SLE reported falls over the prior year.^26^ This may reflect differences in falls measurement across the studies, or geographic or population differences in falls. To our knowledge, there are no other published estimates of fall prevalence among those with SLE, either in or outside of the USA.^7^ However, the overall crude prevalence estimate (24%) in the pooled SLE cohort is similar to a pooled estimate for similarly aged patients with multiple sclerosis (28%),^27^ and the crude estimate among those aged 45 or older (30%) in the pooled SLE cohort is similar to the pooled estimate from patients with another rheumatic disease, rheumatoid arthritis (34%).^28^ Importantly, however, age-standardised and sex-standardised estimates from the pooled SLE cohort did not differ substantially from those in the general US population among those aged 45 or older. While this may reflect no true differences between the SLE and general populations in terms of falls, it may also reflect the availability of data only among the older population for comparison. For example, relatively higher fall prevalence among the younger participants (aged 20–44) would be masked in our analysis. However, data from the Baltimore Longitudinal Study of Aging suggests a fall prevalence of 18% among younger US adults, similar to our crude estimate among younger SLE participants (20%).^29^

Unlike our previous study involving only GOAL participants,^8^ older age was associated with a higher prevalence of falls in our pooled SLE cohort. This may simply be due to greater power to detect age differences in fall prevalence in this larger cohort. Given the substantially different distribution of race/ethnicity between the cohorts, it may also reflect potential effect modification by race/ethnicity (ie*,* lesser effects of age on fall risk in the black subpopulation), which might explain the higher prevalence of falls in GOAL relative to CLUES among the subgroups with lower disease activity and burden. Regardless, the association of older age with higher fall prevalence in the pooled SLE cohort mirrors similar, well-known patterns in the US general population and suggests that older adults should be considered at higher risk for falls than their counterparts, regardless of their SLE status. Interestingly, despite obesity^30^ and low physical activity^31^ being strong risk factors for falls in the general population, we found that, after adjustment, differences in the pooled SLE cohort by these factors were not statistically significant.

However, we also found evidence that SLE-specific and SLE-related factors contribute to falls in this population. Higher SLE activity and greater pain interference were both associated with ~threefold fold higher prevalence of falls, and greater fatigue was associated with ~twofold higher fall prevalence. Lower versus higher perceived cognitive function was associated with two times the reported prevalence of falls, mirroring the general population, in whom shared cognitive and motor pathways are hypothesised.^32^ Pain interference and, especially opioid use, were associated with higher prevalence of falls in the pooled SLE cohort; opioids have been consistently shown to increase fall risk in the general population.^33^ There were also several strong psychosocial correlates of fall prevalence. Both moderate-to-severe versus mild or no depressive symptoms and higher versus lower perceived stress were associated with fall prevalence estimates that were nearly two times as high and only slightly attenuated with adjustment. Some of the effect of depressive symptoms may be due to antidepressant medication use, which is associated with an increased risk of falls in older adults.^34 35^ In fact, in our previous analysis of GOAL data, we found that antidepressant use was associated with 82% higher prevalence of falls,^8^ but these data were not collected in CLUES.

Several potential limitations of our study deserve mention. The 12-month recall period may have introduced misclassification of falls, particularly among those who had more memorable falls (*eg,* associated with a serious injury) or among those with cognitive dysfunction (who might be less likely to remember falls). There is also the possibility of residual confounding, including confounding by use of medications other than antidepressants that increase fall risk, such as antihypertensives and benzodiazepines. Different items measuring fall-related injuries in the pooled SLE cohort data (falls requiring healthcare visits or medical attention) versus BRFSS (falls requiring medical attention or that limited your regular activities for at least a day) likely contributed to relatively lower estimates of injury fall prevalence, since it is likely that many individuals do not or cannot access healthcare during a short fall injury recovery period. Importantly, we did not collect data on the circumstances of the falls in both cohorts. Most falls are due to a combination of intrinsic or predisposing factors (eg*,* balance, strength, dizziness) and extrinsic or precipitating factors (eg*,* slip or trip hazards, footwear, lighting). Understanding the contributions of these factors to patient-reported falls can provide clinicians with individualised fall prevention strategies. BILD and cognitive function were missing for substantial proportions of participants, introducing the possibility of selection bias in these analyses; however, we found similar results in sensitivity analyses using multiple imputation. Finally, although the pooled SLE cohort was large, the results may not be generalisable outside of their respective catchment areas, and some subgroups, particularly those defined by multiple factors (eg*,* sex and race/ethnicity), remained too small for robust analysis.

In conclusion, we found that falls and their recurrence were common in this pooled SLE cohort. While our standardised estimates suggest that our prevalence was similar to that in the US population, we found that factors associated with falls in those with SLE versus the general population may differ, with greater SLE-related disease activity and symptoms associated with a higher prevalence of falls. Given these observations, the clinical assessment of falls, especially for patients with high disease activity, pain, fatigue, depression or cognitive complaints, remains an important component of holistic SLE care. Future studies evaluating the implementation of falls assessment into routine SLE care and the effectiveness of such assessments to prevent poor outcomes, such as fractures, are warranted.

## Supplementary material

10.1136/lupus-2026-002156 online supplemental file 1

## Data Availability

Data are available upon reasonable request.

## References

[1] Arnaud L, Tektonidou MG (2020). Long-term outcomes in systemic lupus erythematosus: trends over time and major contributors. Rheumatology (Oxford).

[2] Izmirly P, Ferucci E, Hersh A (2025). Age-specific Incidence of Systemic Lupus Erythematosus in the United States: A Meta-Analysis of Data from the Centers for Disease Control and Prevention Lupus Registries. Arthritis Care Res.

[3] van den Hoogen LL, Sims GP, van Roon JAG (2015). Aging and Systemic Lupus Erythematosus - Immunosenescence and Beyond. Curr Aging Sci.

[4] Somers EC, Goodrich JM, Wang L (2024). Associations between CD70 methylation of T cell DNA and age in adults with systemic lupus erythematosus and population controls: The Michigan Lupus Epidemiology & Surveillance (MILES) Program. J Autoimmun.

[5] Katz P, Dall’Era M, Plantinga L (2025). Measuring Frailty in Systemic Lupus Erythematosus. Arthritis Care Res (Hoboken).

[6] Katz PP, Andrews J, Yazdany J (2017). Is frailty a relevant concept in SLE?. Lupus Sci Med.

[7] Cho C, Bak G, Richards B (2025). Epidemiology and factors associated with osteoporosis, falls and fractures in patients with chronic inflammatory rheumatic disease: a scoping review. BMJ Open.

[8] Perfect CR, Bowling CB, Lim SS (2025). Falls Among Individuals With Systemic Lupus Erythematosus: An Observational Study. *ACR Open Rheumatol*.

[9] Hochberg MC (1997). Updating the American College of Rheumatology revised criteria for the classification of systemic lupus erythematosus. Arthritis Rheum.

[10] Dall’Era M, Cisternas MG, Snipes K (2017). The Incidence and Prevalence of Systemic Lupus Erythematosus in San Francisco County, California: The California Lupus Surveillance Project. *Arthritis Rheumatol*.

[11] Salazar G, Bauer S, Hoge C (2025). Urinary Incontinence Among Adults With Systemic Lupus Erythematosus. *ACR Open Rheumatol*.

[12] Centers for Disease Control and Prevention (2023). Data from: behavioral risk factor surveillance system survey data. https://www.cdc.gov/brfss.

[13] U.S. Census Bureau (2009). A compass for understanding and using American community survey data: what researchers need to know. https://www.census.gov/content/dam/Census/library/publications/2009/acs/ACSResearch.pdf.

[14] Karlson EW, Daltroy LH, Rivest C (2003). Validation of a Systemic Lupus Activity Questionnaire (SLAQ) for population studies. Lupus.

[15] Yazdany J, Trupin L, Gansky SA (2011). Brief index of lupus damage: A patient‐reported measure of damage in systemic lupus erythematosus. Arthritis Care Res (Hoboken).

[16] Drenkard C, Yazdany J, Trupin L (2014). Validity of a self-administered version of the brief index of lupus damage in a predominantly African American systemic lupus erythematosus cohort. Arthritis Care Res (Hoboken).

[17] Lee PH, Macfarlane DJ, Lam TH (2011). Validity of the International Physical Activity Questionnaire Short Form (IPAQ-SF): a systematic review. Int J Behav Nutr Phys Act.

[18] Northwestern University Introduction to PROMIS. http://www.healthmeasures.net/explore-measurement-systems/promis/intro-to-promis.

[19] Cella D, Choi SW, Condon DM (2019). PROMIS^®^ Adult Health Profiles: Efficient Short-Form Measures of Seven Health Domains. Value Health.

[20] Kroenke K, Strine TW, Spitzer RL (2009). The PHQ-8 as a measure of current depression in the general population. J Affect Disord.

[21] Teresi JA, Ocepek-Welikson K, Kleinman M (2016). Psychometric Properties and Performance of the Patient Reported Outcomes Measurement Information System^®^ (PROMIS^®^) Depression Short Forms in Ethnically Diverse Groups. Psychol Test Assess Model.

[22] Kroenke K, Stump TE, Chen CX (2020). Minimally important differences and severity thresholds are estimated for the PROMIS depression scales from three randomized clinical trials. J Affect Disord.

[23] Warttig SL, Forshaw MJ, South J (2013). New, normative, English-sample data for the Short Form Perceived Stress Scale (PSS-4). J Health Psychol.

[24] Cohen S, Kamarck T, Mermelstein R (1983). A global measure of perceived stress. J Health Soc Behav.

[25] Plantinga L, Tift BD, Dunlop‐Thomas C (2018). Geriatric Assessment of Physical and Cognitive Functioning in a Diverse Cohort of Systemic Lupus Erythematosus Patients: A Pilot Study. Arthritis Care Res (Hoboken).

[26] Yu HJ, Lee WC, Lee KL (2004). Voiding dysfunction in women with systemic lupus erythematosus. Arthritis Rheum.

[27] Giannopapas V, Smyrni V, Kitsos DK (2026). Falls Prevalence and Fear of Falling in Multiple Sclerosis: A Systematic Review and Meta-Analysis. Eur Neurol.

[28] Guo X, Pei J, Wei Y (2023). Prevalence and risk factors of falls in adults with rheumatoid arthritis: A systematic review and meta-analysis. Semin Arthritis Rheum.

[29] Talbot LA, Musiol RJ, Witham EK (2005). Falls in young, middle-aged and older community dwelling adults: perceived cause, environmental factors and injury. BMC Public Health.

[30] G R Neri S, S Oliveira J, B Dario A (2020). Does Obesity Increase the Risk and Severity of Falls in People Aged 60 Years and Older? A Systematic Review and Meta-analysis of Observational Studies. J Gerontol A Biol Sci Med Sci.

[31] Kwok WS, Khalatbari-Soltani S, Dolja-Gore X (2024). Leisure-Time Physical Activity and Falls With and Without Injuries Among Older Adult Women. *JAMA Netw Open*.

[32] Holtzer R, Friedman R, Lipton RB (2007). The relationship between specific cognitive functions and falls in aging. Neuropsychology.

[33] Seppala LJ, van de Glind EMM, Daams JG (2018). Fall-Risk-Increasing Drugs: A Systematic Review and Meta-analysis: III. Others. J Am Med Dir Assoc.

[34] Bender DA, Mulsant BH, Lavretsky H (2026). Risk Factors for Falls in Older Depressed Adults Treated with Bupropion: An Analysis of the OPTIMUM Randomized Clinical Trial. J Gen Intern Med.

[35] White LE, Keedy CA, Xu J (2026). Examining individual serotonin and norepinephrine reuptake inhibitors and fall-related hospitalizations in older adults. *Ment Health Clin*.

