# Supplementary material for: Prevalence of falls in a pooled US cohort of adults with systemic lupus erythematosus: a nested cross-sectional study
Source: Lupus Sci Med. 2026 Aug 17;13(2):e002156. doi: 10.1136/lupus-2026-002156 (PMC13504932; doi:10.1136/lupus-2026-002156)
Supplement: Supplementary file 1 [file lupus-13-2-s001.pdf]

Supplement: Plantinga LC *et al*. Prevalence of Falls in a Pooled U.S. Cohort of Adults with Systemic Lupus Erythematosus: A Nested Cross-Sectional Study

Table of Contents

Supplementary Figure 1. Study design, timeline, and data sources... 2

Supplementary Figure 2. Flow of participants from the two adult systemic lupus erythematosus cohorts into the pooled cohort.. 3

Supplementary Table 1. Assessment of falls in BRFSS, CLUES, and GOAL 4

Supplementary Table 2. Selected characteristics of study participants with systemic lupus erythematosus, by cohort 5

Supplementary Table 3. Unadjusted prevalence of any falls reported in the past year, by cohort and clinical and psychosocial characteristics.. 6

Supplementary Table 5. Prevalence of two or more falls in the prior year among adults with SLE and the U.S. population, aged 45 years or older 8

Supplementary Table 6. Prevalence of any injury fall in the prior year among adults with SLE and the U.S. population, aged 45 years or older 9

Supplementary Figure 1. Study design, timeline, and data sources.

ACS, American Community Survey; APPEAL, Approaches to Positive, Patient-centered Experiences of Aging with Lupus (GOAL ancillary study); BMI, body mass index; BRFSS, Behavioral Risk Factor Surveillance System; BILD, Brief Index of Lupus Damage; CLUES, California Lupus Epidemiology Study; GOAL, Georgians Organized Against Lupus; SLAQ, Systemic Lupus Activity Questionnaire.

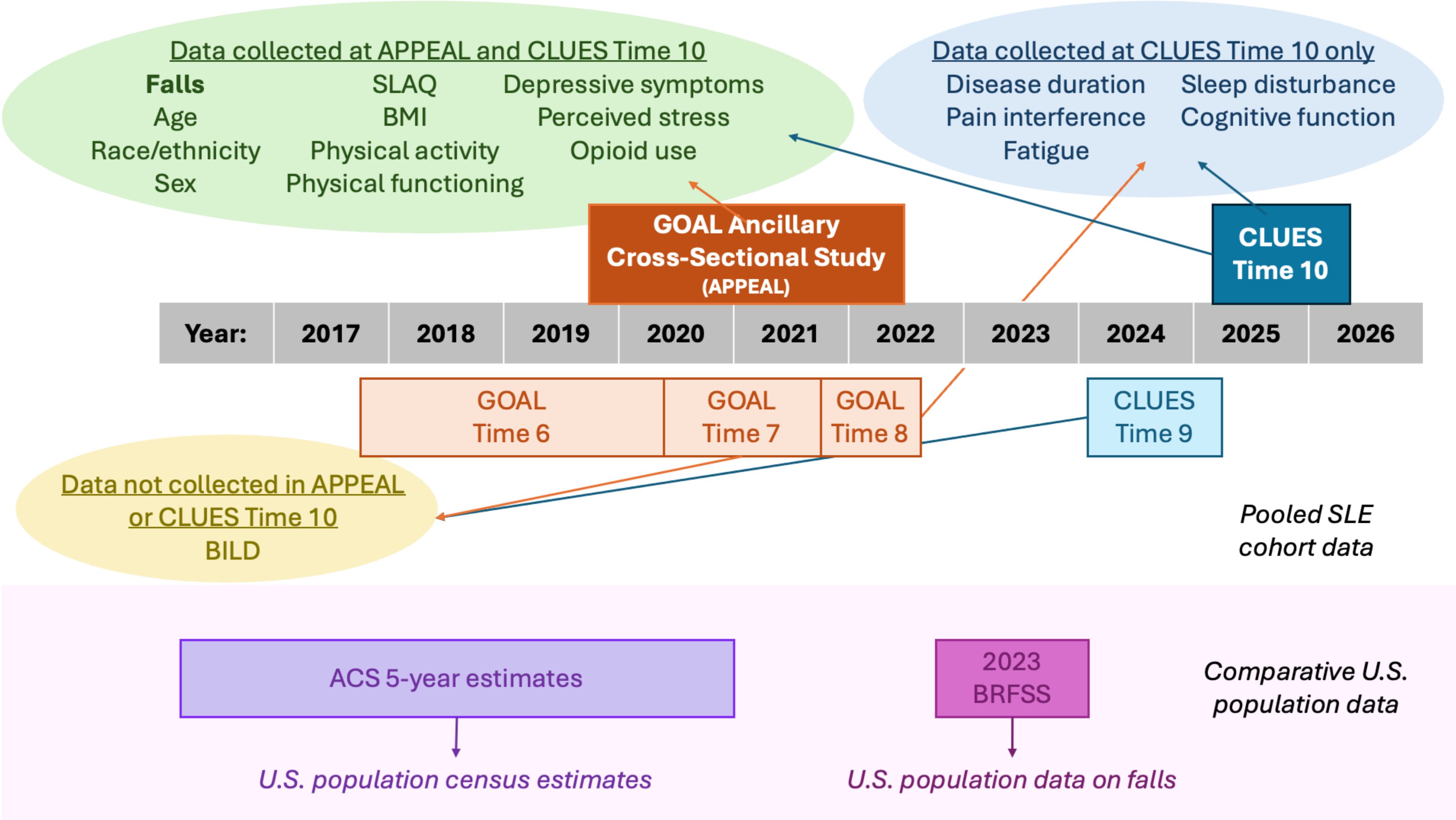

Supplementary Figure 2. Flow of participants from the two adult systemic lupus erythematosus cohorts into the pooled cohort.

CLUES, California Lupus Epidemiology Study; GOAL, Georgians Organized Against Lupus.

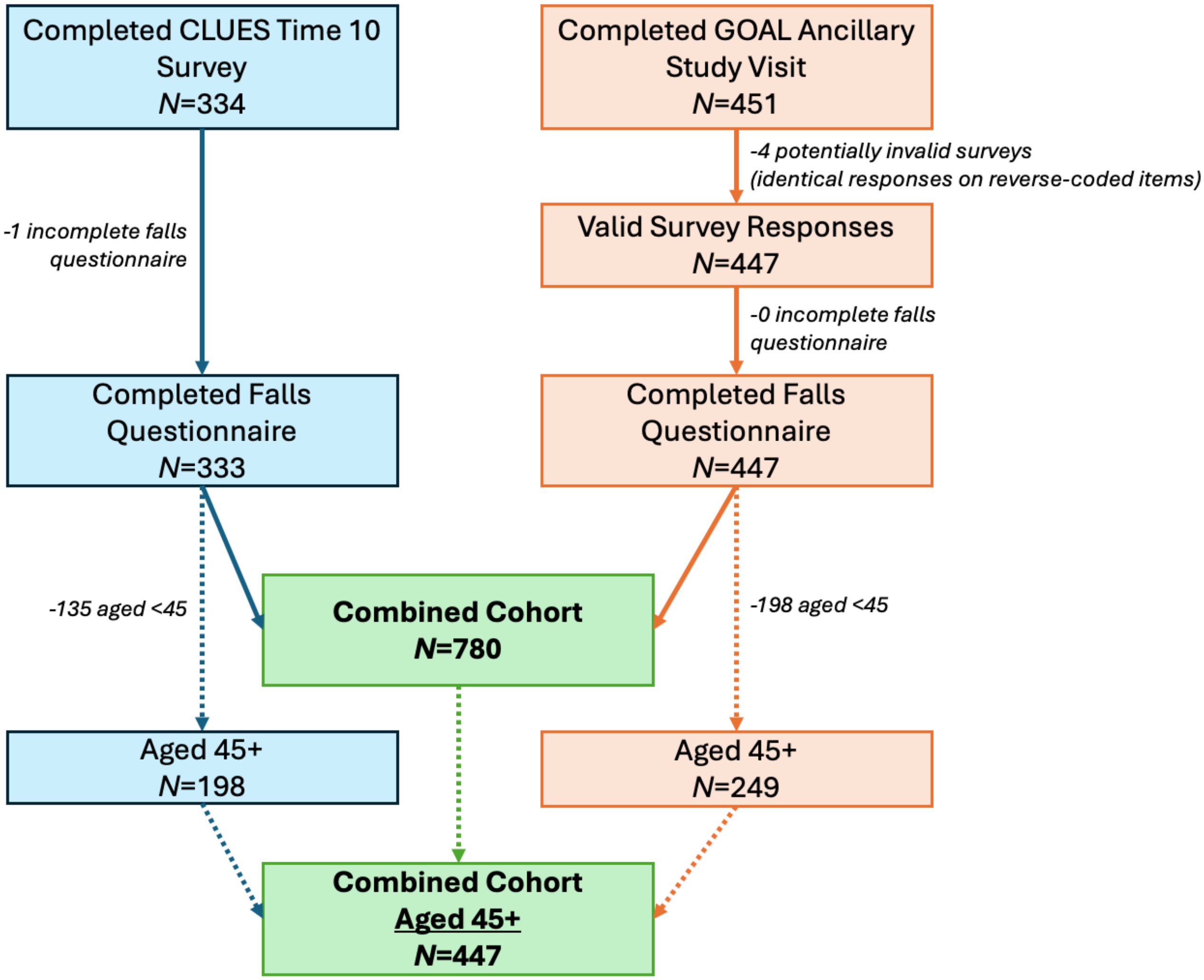

Supplementary Table 1. Assessment of falls in BRFSS, CLUES, and GOAL

Item	BRFSS	CLUES	GOAL
**Administration**	Administered by trained interviewers over the phone (landline and mobile)	Administered by trained interviewers over the phone	Self-administered by participants via REDCap
**Dates**	2023[a]	March 2025-February 2026	October 2019-May 2022
**Any falls in prior year**	---	Have you fallen in the past 12 months? *Possible responses: no, yes, don't know* *[if needed: A fall is an unplanned movement that results in coming to rest on a lower level, such as onto furniture or the floor.]*	Have you fallen in the past 12 months *Possible responses: no, yes*
**Number of falls in prior year**	In the past 12 months, how many times have you fallen? *Possible responses: 1-76, none, don't know*	How many times have you fallen in the past 12 months? *Possible responses: any integer*	How many times have you fallen in the last 12 months? *Possible responses: any integer*
**Injury falls**	How many of these falls caused an injury that limited your regular activities for at least a day or caused you to go see a doctor? *Possible responses: 1-76, none, don't know*	For how many of these falls did you go to the doctor, emergency room, or hospital? *Possible responses: any integer*	How many of your falls in the past 12 months resulted in an injury that required medical attention? *Possible responses: any integer*

BRFSS, Behavioral Risk Factor Surveillance System; CLUES, California Lupus Epidemiology Study; GOAL, Georgians Organized Against Lupus; NHANES, National Health and Nutrition Examination Survey; SP, survey participant.
[a]Exact dates of interviews not available in this de-identified dataset.

Supplementary Table 2. Selected characteristics of study participants with systemic lupus erythematosus, by cohort

	Cohort		
**Characteristic**	**CLUES** ***N*****=333**	**GOAL** ***N*****=447**	***P*****[a]**
Sociodemographic			
Mean (SD) age in years	50.3 (15.1)	46.2 (11.8)	*<0.001*
Age category, *n* (%)			*<0.001*
20-44	135 (40.5)	198 (44.3)	
44-54	70 (21.0)	132 (29.5)	
55-64	51 (15.3)	95 (21.3)	
≥65	77 (23.1)	22 (4.9)	
Sex at birth, *n* (%)			*0.92*
Women	307 (92.2)	410 (91.7)	
Men	26 (7.8)	37 (8.3)	
Race, *n* (%)			*<0.001*
Asian	106 (31.8)	7 (1.6)	
Black	39 (11.7)	364 (81.4)	
Hispanic	78 (23.4)	25 (5.6)	
White	100 (30.0)	38 (8.5)	
Other	10 (3.0)	13 (2.9)	
Clinical, physical, and psychosocial			
Median (IQR) disease duration, years	24.0 (14.0, 32.0)	15.0 (9.6, 22.4)	*<0.001*
Median (IQR) SLAQ score	7.0 (3.0, 12.0)	13.0 (7.0, 19.0)	*<0.001*
Median (IQR) BILD score	2.0 (1.0, 4.0)	2.0 (1.0, 4.0)	*0.09*
Mean (SD) BMI, kg/m^2	26.6 (6.2)	30.1 (8.2)	*<0.001*
Obese, *n* (%)			*<0.001*
Yes	86 (25.9)	206 (47.2)	
No	246 (74.1)	230 (52.8)	
Physical activity, *n* (%)			*<0.001*
Low	67 (20.1)	325 (73.9)	
Moderate/High	266 (79.9)	115 (26.1)	
Mean (SD) physical function T-score	47.3 (9.7)	41.5 (9.7)	*<0.001*
Depression, *n* (%)			*0.92*
Moderate or severe	47 (14.1)	63 (14.7)	
None or mild	286 (85.9)	376 (85.3)	
Median (IQR) perceived stress score	5.0 (2.0, 7.0)	7.0 (4.0, 8.0)	*<0.001*
Mean (SD) pain interference T-score	52.3 (9.2)	57.4 (10.5)	*<0.001*
Mean (SD) fatigue T-score	53.5 (10.5)	56.9 (11.5)	*<0.001*
Mean (SD) sleep disturbance T-score	52.3 (8.9)	55.8 (11.6)	*<0.001*
Mean (SD) cognitive function T-score	45.3 (9.6)	48.5 (11.2)	*0.004*
Opioid pain medications, *n* (%)			*0.48*
Yes	41 (12.4)	47 (10.5)	
No	290 (87.6)	400 (89.5)	

BMI, body mass index; CLUES, California Lupus Epidemiology Study; GOAL, Georgians Organized Against Lupus; IQR, interquartile (25th-75th percentile) range; SLAQ, Systemic Lupus Activity Questionnaire (range, 0-44; 44 is maximum activity). Missing values: ethnicity (GOAL, *n*=1; CLUES, *n*=1), BILD score (CLUES, *n*=44), BMI/obesity (GOAL, *n*=11; CLUES, *n*=1), physical activity (GOAL, *n*=7), depressive symptoms score (GOAL, *n*=17), and perceived stress score (GOAL, *n*=2), pain interference score (GOAL, *n*=7), fatigue score (GOAL, *n*=1), sleep disturbance score (GOAL, *n*=145), cognitive function score (GOAL, *n*=309), opioid pain medications (CLUES, *n*=2).
[a]Comparing cohort values using *t*, Wilcoxon rank-sum, chi-square, or Fisher's exact test, as appropriate.

Supplementary Table 3. Unadjusted prevalence of any falls reported in the past year, by cohort and clinical and psychosocial characteristics

	Percentage falling in prior year (95% CI)		
**Characteristic**	**CLUES**	**GOAL**	***P*_interaction**
Disease duration			
<10 years	11.8 (4.9–24.6)	29.3 (21.6–38.3)	*0.2*
≥10 years	21.3 (16.7–26.6)	31.3 (26.3–36.7)	
Disease activity			
<median (=9)	10.7 (7.2–15.5)	17.9 (12.4–25.1)	*0.04*
≥median[b]	41.4 (31.7–51.8)	37.5 (31.9–43.3)	
Disease damage			
<median (=2)	12.1 (7.6–18.5)	27.0 (21.3–33.6)	*0.04*
≥median[b]	29.5 (22.1–38.2)	33.9 (28.0–40.4)	
Obesity			
No	19.9 (15.2–25.6)	25.7 (20.2–31.9)	*0.2*
Yes	19.8 (12.3–30.0)	36.4 (29.9–43.4)	
Physical function			
High-average (T-score >40)	13.3 (9.5–18.2)	17.9 (13.1–23.9)	*0.4*
Low (T-score ≤40)	41.6 (30.6–53.4)	42.1 (35.8–48.7)	
Physical activity			
Low	22.4 (13.5–34.5)	33.8% (28.8–39.3)	*0.4*
Moderate-High	19.2 (14.7–24.5)	23.5% (16.3–32.5)	
Depression			
Moderate or severe	40.4 (26.7–55.7)	39.7 (27.8–52.8)	*0.08*
None or mild	16.4 (12.4–21.4)	29.4 (24.9–34.4)	
Perceived stress			
<median (=5)	15.3 (10.9–21.1)	23.9 (18.0–30.9)	*0.6*
≥median[b]	27.4 (20.0–36.3)	35.1 (29.4–41.2)	
Pain interference			
High (T-score ≥60)	46.7 (35.2–58.5)	42.8 (35.9–49.9)	*0.02*
Low-average (T-score <60)	12.0 (8.4–16.8)	21.3 (16.4–27.2)	
Fatigue			
High (T-score ≥60)	38.9 (29.0–49.8)	39.3 (32.3–46.8)	*0.03*
Low-average (T-score <60)	12.8 (9.0–17.8)	24.3 (19.4–30.1)	
Sleep disturbance			
High (T-score ≥60)	29.4 (17.9–44.0)	37.6 (28.7–47.4)	*0.9*
Low-average (T-score <60)	18.1 (13.9–23.2)	25.4 (19.5–32.2)	
Cognitive function			
High-average (T-score >40)	14.2 (10.2-19.2)	30.7 (22.1–40.8)	*0.1*
Low (T-score ≤40)	38.0 (27.5-49.6)	43.2 (27.5–60.4)	
Opioid medication use			
Yes	41.5 (26.7–57.8)	61.7 (46.4–75.1)	*0.6*
No	16.9 (12.9–21.8)	27.0 (22.8–31.7)	

Supplementary Table 4. Prevalence of any fall in the prior year among adults with SLE and the U.S. population, aged 45 years or older

	Estimated prevalence of any fall in the prior 12 month, % (95% CI)				
	Pooled SLE cohort participants aged ≥45 (N=447)				
**Population**	**Crude**	**Age-standardized**	**Age- and sex-standardized[a]**	**Age-, sex-, and race/ethnicity-standardized[a]**	**U.S. population aged ≥45[b]**
Overall	30.0 (25.9-34.4)	28.3 (23.6-33.4)	28.0 (18.9-39.3)	22.9 (16.2-31.3)	24.4 (24.1-24.8)
Age					
45-54	26.7 (21.1-33.3)	---	26.0 (16.6-38.2)	15.2 (9.3-23.8)	19.5 (18.8-20.2)
55-64	29.5 (22.6-37.4)	---	19.9 (13.0-29.5)	19.5 (11.4-31.2)	24.0 (23.3-24.7)
≥65	37.4 (28.4-47.3)	---	43.3 (21.9-67.9)	40.8 (27.5-55.5)	27.8 (27.3-28.3)
Sex					
Women	30.7 (26.4-35.3)	29.5 (24.5-35.0)	29.5 (24.5-35.0)	30.7 (21.9-41.1)	26.4 (25.9-26.9)
Men	21.2 (10.4-38.3)	15.3 (6.5-32.0)	15.3 (6.5-32.0)	10.4 (3.2-28.9)	22.2 (21.7-22.7)
Race/ethnicity					
Asian	8.2 (3.1-19.9)	7.5 (2.3-21.7)	4.5 (1.4-14.0)	4.5 (1.4-14.0)	12.5 (10.3-15.1)
Black	32.3 (26.5-38.7)	32.2 (25.8-39.3)	28.4 (17.3-42.8)	28.4 (17.3-42.8)	22.0 (20.9-23.2)
Hispanic	26.9 (16.6-40.5)	25.9 (13.9-42.9)	16.4 (9.2-27.7)	16.4 (9.2-27.7)	25.8 (25.4-26.2)
White	37.6 (29.0-47.1)	31.3 (21.1-43.7)	37.8 (29.7-46.6)	37.8 (29.7-46.6)	31.2 (29.3-33.2)

[a]Excluding the stratification variable (e.g., age-stratified estimates are standardized for sex, race/ethnicity).
[b]Weighted estimates from the 2023 Behavioral Risk Factors Surveillance Survey.

Supplementary Table 5. Prevalence of two or more falls in the prior year among adults with SLE and the U.S. population, aged 45 years or older

	Estimated prevalence of two or more falls in the prior 12 month, % (95% CI)				
	Pooled SLE cohort participants aged ≥45 (N=447)				
**Population**	**Crude**	**Age-standardized**	**Age- and sex-standardized[a]**	**Age-, sex-, and race/ethnicity-standardized[a]**	**U.S. population aged ≥45[b]**
Overall	18.3 (15.0-22.2)	16.9 (13.2-21.4)	16.0 (8.9-26.9)	11.4 (7.8-16.4)	12.8 (12.5-13.0)
Age					
45-54	18.3 (13.6-24.3)	---	15.7 (9.0-26.1)	10.9 (6.0-19.1)	10.5 (10.0-11.0)
55-64	15.8 (19.7-22.6)	---	12.2 (6.3-22.4)	8.8 (3.7-19.6)	13.3 (12.8-13.8)
≥65	22.2 (15.1-31.5)	---	23.5 (8.6-50.0)	27.4 (17.3-40.5)	13.8 (13.4-14.3)
Sex					
Women	18.8 (15.3-22.9)	17.5 (13.6-22.3)	17.5 (13.6-22.3)	17.3 (10.9-26.4)	13.3 (12.9-13.7)
Men	12.1 (4.6-28.2)	9.8 (3.1-27.0)	9.8 (3.1-27.0)	2.3 (0.9-6.0)	12.1 (11.7-12.5)
Race/ethnicity					
Asian	6.1 (2.0-17.4)	5.5 (1.3-20.3)	3.5 (0.8-13.6)	3.5 (0.8-13.6)	4.4 (3.3-5.9)
Black	19.9 (15.2-25.6)	19.5 (14.4-25.9)	12.3 (7.1-20.7)	12.3 (7.1-20.7)	11.5 (10.7-12.4)
Hispanic	15.4 (7.9-27.9)	13.3 (5.5-20.9)	8.2 (3.3-18.6)	8.2 (3.3-18.6)	13.3 (13.0-13.6)
White	23.9 (16.8-32.8)	19.3 (11.6-30.4)	32.1 (24.7-40.5)	32.1 (24.7-40.5)	19.2 (17.6-21.0)

[a]Excluding the stratification variable (e.g., age-stratified estimates are standardized for sex, race/ethnicity).
[b]Weighted estimates from the 2023 Behavioral Risk Factors Surveillance Survey.

Supplementary Table 6. Prevalence of any injury fall in the prior year among adults with SLE and the U.S. population, aged 45 years or older

	Estimated prevalence of any injury fall in the prior 12 month, % (95% CI)				
	Pooled SLE cohort participants aged ≥45 (N=447)				
**Population**	**Crude**	**Age-standardized**	**Age- and sex-standardized[a]**	**Age-, sex-, and race/ethnicity-standardized[a]**	**U.S. population aged ≥45[b]**
Overall	10.7 (8.1-14.0)	10.1 (7.2-13.9)	6.9 (4.2-11.0)	6.1 (3.2-11.2)	10.0 (9.8-10.3)
Age					
45-54	8.9 (5.7-13.7)	---	10.5 (4.7-21.9)	5.0 (2.1-11.5)	8.6 (8.2-9.1)
55-64	11.0 (6.8-17.2)	---	6.2 (3.9-9.7)	7.4 (2.7-18.7)	10.3 (9.8-10.8)
≥65	14.1 (8.5-22.5)	---	7.5 (4.6-12.1)	4.0 (2.5-6.5)	10.7 (10.4-11.1)
Sex					
Women	11.1 (8.4-14.5)	10.5 (7.4-14.7)	10.5 (7.4-14.7)	10.0 (5.0-19.0)	12.2 (11.8-12.6)
Men	6.1 (1.5-21.3)	5.5 (1.5-18.8)	5.5 (1.5-18.8)	1.3 (0.3-4.7)	7.6 (7.3-7.9)
Race/ethnicity					
Asian	2.0 (0.3-13.1)	3.5 (0.5-20.6)	2.4 (0.4-14.5)	2.4 (0.4-14.5)	4.3 (3.0-6.1)
Black	10.6 (7.2-15.0)	10.9 (7.1-16.3)	7.2 (3.8-13.4)	7.2 (3.8-13.4)	9.6 (8.8-10.4)
Hispanic	5.8 (1.9-16.4)	9.1 (2.9-25.0)	5.9 (1.9-16.7)	5.9 (1.9-16.7)	9.3 (8.5-10.1)
White	17.4 (11.4-25.7)	11.6 (5.8-22.0)	8.0 (3.5-17.2)	8.0 (3.5-17.2)	10.4 (10.0-10.6)

[a]Excluding the stratification variable (e.g., age-stratified estimates are standardized for sex, race, and ethnicity).
[b]From the 2023 Behavioral Risk Factors Surveillance Survey.
